# The Thermal Characteristics, Sorption Isotherms and State Diagrams of the Freeze-Dried Pumpkin-Inulin Powders

**DOI:** 10.3390/molecules27072225

**Published:** 2022-03-29

**Authors:** Anna Stępień, Mariusz Witczak, Teresa Witczak

**Affiliations:** Department of Engineering and Machinery for Food Industry, Faculty of Food Technology, University of Agriculture, Balicka 122 Street, 30-149 Kraków, Poland; rrwitcza@cyf-kr.edu.pl (M.W.); teresa.witczak@urk.edu.pl (T.W.)

**Keywords:** glass transition, sorption isotherms, state diagram

## Abstract

Powders based on plant raw materials have low storage stability due to their sorption and thermal properties and generate problems during processing. Therefore, there is a need to find carrier agents to improve their storage life as well as methods to evaluate their properties during storage. Water adsorption isotherms and thermal characteristics of the pumpkin powder with various inulin additions were investigated in order to develop state diagrams. Differential scanning calorimetry (DSC) was used to obtained glass transition lines, freezing curves and maximal-freeze-concentration conditions. The glass transition lines were developed using the Gordon–Taylor model. Freezing data were modeled employing the Clausius–Clapeyron equation and its development–Chen model. The glass transition temperature of anhydrous material (Tgs) and characteristic glass transition temperature of maximum-freeze-concentration (Tg′) increased with growing inulin additions. Sorption isotherms of the powders were determined at 25 °C by the static-gravimetric method and the experimental data was modeled with four different mathematical models. The Peleg model was the most adequate to describe the sorption data of the pumpkin–inulin powders. Guggenheim-Anderson-de Boer (GAB) monolayer capacity decreased with increasing inulin concentration in the sample.

## 1. Introduction

The shelf life of powdered food depends on many factors, but the two most important of them are the balance of moisture content and the glass transition temperature. Especially susceptible to negative changes during storage and processing are raw vegetable materials, including pumpkin pulp selected as a subject of this study. One of the methods of improving the storage properties of food powders is the addition of high molecular weight substances; therefore, in this publication we focused on inulin, which is additionally a health-promoting component due to its prebiotic effect. A tool whose usefulness in assessing the storage stability of powders of varying composition are state diagrams that combine the concepts of water activity and glass transition. 

The most commonly tool used to graphically represent the relationship between equilibrium moisture content and water activity is the adsorption isotherm. Its shape characterizes sorption into three main regions. The nature of the sorption curve and the parameters of the models used for its determination provide information on the interaction between water and the components of the food product at the thermodynamic equilibrium state. Knowledge of the sorption characteristics of foodstuff is therefore applicable to many processes in the food industry such as packaging, drying, determining physicochemical and microbiological stability, and designing drying processes. Due to the complex composition and varied interactions between components of biological materials, the sorption isotherms of a given product must be determined based on experimental studies. The key parameter, from the point of view of the water activity concept, is the monolayer water content, determined from appropriate sorption models, which corresponds to the material moisture at which it is most stable [1].

During the production, storage and distribution of foodstuffs, many physical and chemical changes may take place. Therefore, the knowledge of phase transitions in food materials is of great importance in determining its quality, stability control and designing technological processes. The glass transition is a characteristic phenomenon of amorphous materials, which include most food products undergoing technological processing (drying, baking, freeze-drying, extrusion). The glass transition is defined as the temperature (Tg) at which a material undergoes a transformation from the glassy to the rubbery state. From the point of view of material stability, the glassy state is more desirable due to the fact that it has a higher viscosity and lower molecular mobility, which ensures that the possibility of occurrence of physicochemical and biological changes is limited. The development of glass transition is associated with a number of undesirable effects in the material such as sticking, crystallization and agglomeration. Therefore, one of the challenges in the area of production of foods susceptible to glass transition is to prevent its occurrence. One way to increase the Tg value of a material is by the addition of high molecular weight polymers [2,3]. Starch, maltodextrin or Arabic gum, among others, are used industrially to raise the glass transition temperature of food products [4].

A state diagram of a food is defined as a map of different physical food states as a function of dry matter or water content and temperature. The main elements of the state diagram are a freezing curve and a glass transition line, and they are used to calculate the maximal-freeze-concentration point. The experimental data used to plot the state diagram are obtained by calorimetric analysis. Knowledge of the parameters determined from the state diagram can be useful during controlling the quality and stability of fresh, frozen and dried foods and in designing technological processes [5]. The suitability of the state diagram has been proven for many plant products such as mango [6], camu-camu [7] or colostral whey powders [8]. In our previous studies, we have demonstrated that the state diagram can also be used to predict the stability of binary systems involving pumpkin powder with maltodextrin [9]. The next step of our project involved investigating the impact of eliminating starch hydrolysate in exchange for a carrier with health-promoting properties.

Pumpkin is a seasonal vegetable that is used in the food industry mainly in processed form. The most popular pumpkin-based products include juices, purees and jams. In addition, it has been shown that pumpkin flour can be used as an ingredient in gluten-free pastas, breads, and baked products. It is a rich source of dietary fiber, vitamin C, and antioxidants [10,11]. Inulin is a storage carbohydrate found in many plant materials such as chicory, onion, garlic or Jerusalem artichoke tubers. From the chemical point of view it is linear fructan consisting of fructose units chains linked by (2-1)-β-D-fructosyl-fructose bonds with a terminal single glucose unit. Due to bonds that are not digested by human amylase, inulin is a soluble fiber fraction [12]. It has been proven that regular consumption of small doses of inulin brings a number of health benefits such as the simulation of growth and development of the desired microflora in the colon and reduction of bacterial pathogenesis, the support of digestion and prevention of the colon carcinogenesis, the reduction of the triglycerides level in the blood, and the and enhancement of the absorption of calcium and magnesium [13]. Due to its physico-chemical properties, inulin is widely used in the food industry as a sweetener, fat mimetic, textural, gelling and thickening agent [14].

Fructans are increasing in popularity among food producers and consumers because of their demonstrated beneficial prebiotic effect on human health. There are many reports in the literature about the possibility of using inulin as a functional food additive. Saavedra-Leos et al. [15] also presented the possibility of using fructan as a carrier in the spray drying of orange juice. However, there is a lack of knowledge about the use of inulin as an alternative to popular polymers, applied during the plant material drying process in order to increase the glass transition temperature and reduce hygroscopicity of the final powder.

Therefore, the main objective of the present study was to determine the critical storage parameters related to vitreous transition from state diagrams for model systems containing pumpkin powder and inulin. The study considered vitreous transition values for powders for materials stored frozen (Tg′ and Tg‴) and dried (Tg) dry matter contents. Furthermore, the effect of different inulin content on the sorption properties of the powders was also investigated, with particular focus on the monolayer capacity which, according to the concept of water activity, corresponds to the optimum moisture content of the material. Based on the results obtained, it was also evaluated as to whether inulin can be an alternative polymeric carrier as a substitute for commonly used substances such as maltodextrin or starches. 

## 2. Materials and Methods

### 2.1. Raw Material

Pumpkin (Cucurbita maxima) was obtained from plantation of Department of Raw Material and Processing of Fruit and Vegetables, University of Agriculture in Cracow. Before analyses pumpkin peel and seeds were removed. In the research only edible part of fruit was used. Inulin HPX (high performance inulin) with degree of polymerization value ≥ 23 was used (Beneo Orafti^®^, Mannheim, Germany).

### 2.2. Powders Preparation

Pumpkin powders with 25 (P0.75:I0.25), 50 (P0.5:I0.5) and 75% (P0.25:I0.75) of inulin addition were prepared. As a reference, pure pumpkin powder (P1:I0) from our previous study [9] was used. The final composition of powders containing inulin and pumpkin pulp was determined considering the dry weight of the raw materials. The proper amount of the peeled pumpkin flesh and inulin were weighted and mixed to homogenous pulp using a kitchen blender, and samples were frozen in thin layers on Petri dishes at −20 °C for two days. Next, samples were lyophilized under pressure equal 0.035 mbars for 48 h using Alpha 1-2LD plus Christ (Newtown, UK) equipment. Dry samples were milled and sieved in order to obtain powders with the same granules fraction.

### 2.3. Adsorption Isotherms

Sorption isotherms at 25 °C were determined by the static-gravimetric method at 25 °C. The saturated salt solutions LiCl, CH_3_COOK, MgCl_2_, K_2_CO_3_, Mg(NO_3_)_2_, NaNO_2_, NaCl, KCl were prepared in desiccators in order to provide water activity values of 0.11, 0.23, 0.33, 0.43, 0,53, 0.65, 0.75 and 0.86, respectively. To determine equilibrium moisture content data, triplicate powder samples (about 1 g) were weighted into a Petri dish and stored above the saturated salt solutions. In desiccators with water activity values higher than 0.65, crystalline thymol was placed to prevent the microbial growth in the material. Samples were weighted periodically during equilibration until there was not more than 0.001 g difference in weight between measurements. The relationship between the water activity and equilibrium moisture content was modeled using four mathematical models: 

Brunauer–Emmett–Teller (BET) [16]:

(1) $$\mathrm{Xw}=\frac{{\mathrm{XmCa}}_{w}}{\left(1-a_{w}\right)(1+\left(C-1\right)a_{w}}$$

Guggenheim-Anderson-de Boer (GAB) [17]:

(2) $$\mathrm{Xw}=\frac{{\mathrm{XmCKa}}_{w}}{1-{\mathrm{Ka}}_{w}(1-\left(C+1\right){\mathrm{Ka}}_{w}}$$

Peleg [18]:

(3) $$\mathrm{Xw}={\mathrm{Aa}}_{w}^{B}+{\mathrm{Da}}_{w}^{E}$$

Lewicki [19]:

(4) $$\mathrm{Xw}=F\left[\frac{1}{{\left(1-a_{w}\right)}^{G}}-\frac{1}{1+a_{w}^{H}}\right]$$

where: Xw is equilibrium moisture content (g H_2_O/g solids), Xm is monolayer moisture content (g H_2_O/g solids), a_w_ is water activity, K, C, A, B, D, E, F, G and H are models constants.

The fitting models to the experimental data were performed using Statistica (StatSoft, Inc., Tulsa, OK, USA) software version 13.3. The parameters of the models were calculated by using non-linear last square regression with the Lavenberg-Marquadt algorithm. The root mean square (RMS) and coefficient of determination (R^2^) were estimated in order to evaluate the ability of each model to express experimental data.

### 2.4. Differential Scanning Calorimetry (DSC)

The thermal characteristics were measured by using a differential scanning calorimeter DSC 204F1 Phoenix (Netzsch, Germany). An instrument was calibrated using a multipoint method (Hg, In, Sn, Bi, Zn and CsCl). All thermal effects and constants were determined on thermograms by using the Proteus Analysis software (Netzsch, Germany).

#### 2.4.1. Glass Transition of Samples Containing Unfreezable Water

About 10 milligrams of samples with different moisture content were hermetically closed in aluminum pans and analyzed at DSC temperature program from −80 to 200 °C with heat rate of 5 °C/min. A double scanning program was used for each sample in order to reduce the enthalpy relaxation of the amorphous samples, which occurred in the first scan. An empty aluminum pan was used as a reference sample. A nitrogen agent was used as a carrier gas. All analysis was done in duplicate. The glass transition temperature was determined from heating thermograms as an initial point, mid-point and end-point of transition. The mid-point (Tg) was taken as the characteristic temperature of the glass transition value.

#### 2.4.2. Glass Transition of Samples Containing Freezable Water

In order to obtain samples with higher moisture content (30–90% of moisture), proper amounts of distilled water were added to the powders. About 10 milligrams of each sample were closed in an aluminum pan and stored for a few days in order to equilibrate. The measurement procedure based on the methodology presented by Singh & Roos [20] was carried out for all samples. In the first part of the thermal analysis, samples were cooled to −80 °C and reheated to 20 °C without annealing in order to estimate the onset temperature of apparent maximal-freeze-concentration melting Tm′. Next, annealing was performed: samples were cooled to −80 °C and equilibrated for 10 min. Then pans were heated to Tm′–1 temperature, cooled and equilibrated again and scanned from −80 to 50 °C to determine actual Tm′ and Tf values. The intersection of the linear base line of the endotherm with the left side of the endotherm was considered as a Tm′. The peak of the endotherm was estimated as the freezing point (Tf).

### 2.5. Glass Transition Line and Freezing Curve Modeling

All calculations were performed by using nonlinear least square regression with the Lavenberg-Marquardt algorithm in Statistica 13.3 (StatSoft, Inc., Tulsa, OK, USA) software. The glass transition temperature curve was modelled using the Gordon–Taylor equation [21]:

(5) $$\;\mathrm{Tg}=\frac{{\mathrm{Tg}}_{s}\mathrm{Xs}+{\mathrm{kTg}}_{w}\mathrm{Xw}}{\mathrm{Xs}+\mathrm{kXw}}$$

where: Tg–glass transition temperature experimental data [°C]; Tg_s_–model parameter corresponding to glass transition temperature of dry matter [°C]; Tg_w_–glass transition temperature of pure water −135 °C; Xw–water mass ratio; Xs–mass fraction of solids [kg dry solid/kg sample]; k–the Gordon–Taylor parameter.

The Clausius–Clapeyron Equation (8) [22] and the Chen [23] model were used to model the freezing curve:

(6) $$\mathrm{Tf}=-\frac{\beta }{\lambda w}\ln\left[\frac{1-\mathrm{Xs}}{1-\mathrm{Xs}+E*\mathrm{Xs}}\right]$$

(7) $$\mathrm{Tf}=-\frac{\beta }{\lambda w}\ln\left[\frac{1-\mathrm{Xs}-B*\mathrm{Xs}}{1-B*\mathrm{Xs}+E*\mathrm{Xs}}\right]$$

where: Tf–freezing point of the sample [°C]; Tw–freezing point of the water [°C]; β–molar freezing point constant of water [1860 kg K/kg⋅mol]; λw–molecular mass of water; Xs–mass fraction of solids [kg dry solid/kg sample]; E–molecular mass ratio of water to solid [λw/ MW], B–ratio of unfreezable water of the total solid content.

## 3. Results and Discussion

### 3.1. Water Sorption Isotherms

**Figure 1:**
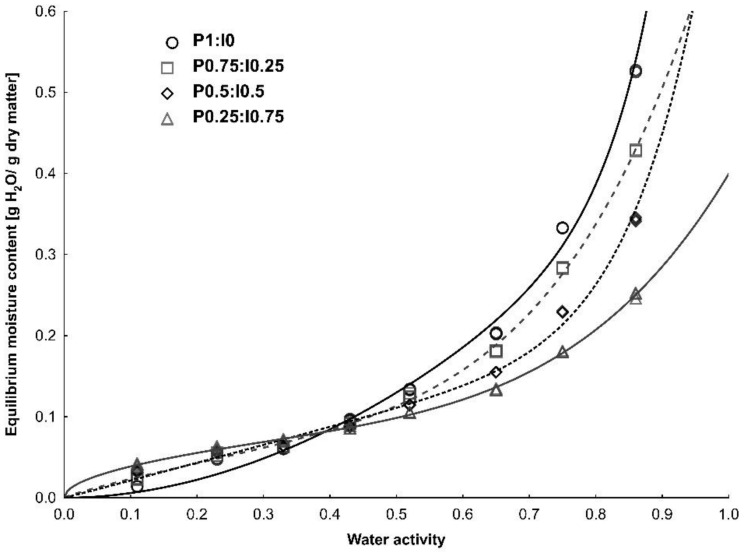
Adsorption isotherms of freeze dried pumpkin powder produced with different inulin additions fitted by the Peleg model.

The water adsorption isotherms of freeze-dried pumpkin powders with various inulin additions, adjusted by Peleg model, are presented in Figure 1. They express the amount of adsorbed moisture (Xw) as a function of water activity in each of the studied powders. It was found that the curve of pure pumpkin powder was J-shaped (type III), which is characteristic of many fruits rich in low molecular weight sugars [24]. The addition of the inulin resulted in the curve shape changing into a sigmoidal type II (according to ) classification. A similar trend was reported for pumpkin powder with maltodextrin additions [9] and avocado powders produced with inulin as a carrier [25]. The sigmoidal curve shape is the most common isotherm type for food products and it is characteristic for materials containing high molecular weight substances such as polysaccharides or proteins [24]

All plotted curves showed an increase in equilibrium moisture content with increasing water activity. At a lower water activity range, powders tend to have a low affinity for adsorbing water, but above critical a_w_ value, materials absorb moisture intensively, which can result in the dissolution of soluble components on the powders′ surfaces and the liquid bridge formation. This behavior is characteristic of crystalline materials [26]. At the first adsorption region, the lowest Xw value were determined for pure pumpkin powder and the highest for the sample containing 75% of the inulin addition. At the higher water activity range this trend was reversed. The changes in hygroscopicity can be explained by the fact that the addition of a high molecular weight polymer to the pumpkin flesh changed the balance between hydrophobic and hydrophilic sites, promoting less adsorbed water at higher water activities. The polymer matrix may also undergo structural changes during the sorption process due to swelling [27]. In the literature there are numerous mathematical models used to describe the sorption process in food products. In our study, experimental data obtained for pumpkin-inulin powders were described by four of them: the BET, GAB, Peleg and Lewicki models. The values of the coefficient of determination and the root mean square error were calculated in order to select the equation best fitted to the raw sorption data. Taking into account that the tested materials were powders including various compositions and variable ratios of individual components, models with R^2^ higher than 0.900 and RMS lower than 15% can be considered adequate and acceptable. The estimated parameters of the mathematical models and the coefficients of determination and root mean square values are presented in Table 1.

The BET and GAB models share two parameters, and based on the same physical adsorption theory are the most popular equations used to describe the sorption data of food products. In almost all cases, the BET model application is limited to a water activity range lower than 0.45, while GAB describes the sorption behavior in a wide range of a_w_ (0–0.9) [28]. In our research, experimental sorption data for the BET model was limited to 0.52 and for GAB models to 0.75 in order to obtain proper goodness of the fit. Through the BET and GAB equations, it is possible to determine the most important shelf-life parameter (Xm) corresponding to the monolayer capacity. According to water activity concept, food products are the most stable when their moisture content is equal to the Xm value [1]. The monolayer moisture content expresses the sorption capacity of the adsorbent and the availability of the active sorption sites [29]. Xm values of pumpkin with different inulin additions calculated for the BET model varied between 0.053 and 0.100 g water/g solids and were in the range 0.057–0.079 g water/g solids for GAB. For both cases, a decrease of Xm with increasing inulin content in the sample was observed. Nevertheless, due to a wider range of experimental data, the results obtained using the GAB model can be considered more representative. A decrease of the monolayer capacity influenced by high molecular weight polymer addition was also reported for pumpkin produced with maltodextrin [9], pineapple [27] and noni [4], with the addition of malodextrins. As is common for the BET and GAB models, sorption energy constant C determines the strength of the binding water molecules to the primary binding sites on the material surface. Water molecules bond in the monolayer, and the binding sites on the surface of the product become stronger with increasing C values [30]. The values of this parameter calculated for the pumpkin-inulin systems from the GAB model, which considers a wider range of data, varied from 2.36 to 16.52. However, it was observed that regardless of the model used for the calculations, the value of C increased with the increasing inulin content in the sample. This allows us to conclude that the presence of fructan in powdered pumpkin resulted in an increased strength in bonding in the monolayer and between water molecules in the material. A similar trend was noted in our previous study in which maltodextrin was added to pumpkin [9]. According to Lewicki [31], the K determined from the GAB model for food materials should be in the following range: 0 < K <1. Apart from one exception, the calculated values for the tested samples do not meet this assumption, and they varied between 0.919 and 1.050. The K constant expresses the interaction between water molecules and the adsorbent in the multilayer. In general, a K value equal to 1 indicates that the heat of evaporation for the multilayers is the same as that for pure water. Overestimated values of the K parameter may be related to the fact that isotherms of powders with decreasing inulin content became less sigmoidal. A similar tendency was observed for pumpkin with maltodextrin [9] and for other foodstuffs [32,33].

The four-parameter and purely empirical Peleg model is assumed to describe experimental data of food materials as well or better than the GAB model, over a wide range of water activities [28]. Its suitability for describing sorption data has been demonstrated for, among others, walnuts [34], pistachio [35], dried tea [36] and apricots [37]. In our study, the correlation coefficient values obtained using the Peleg model for all powders were higher than the other three equations used, and ranged from 0.998 to 0.999. Slightly lower R^2^ values (0.995 to 0.998) were calculated using the Lewicki three-parameter model. It was observed that, independently of the equation used, the highest values of the mean square error were calculated for the sample containing pumpkin without inulin addition, which may be related to the different mechanism of water binding and the different shape of the isotherm itself. All tested models adequately described the sorption data, as noted by R^2^, which ranged from 0.966 to 0.999, and RMS, which ranged from 2.81 to 14.11%.

### 3.2. Glass Transition Temperature

**Figure 2:**
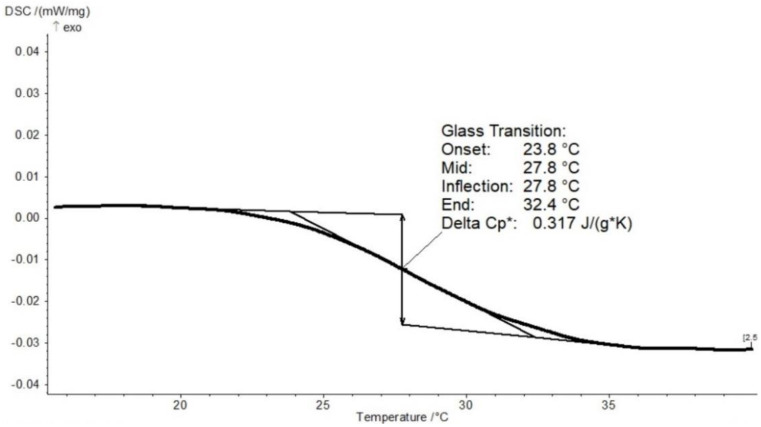
DSC profile of freeze dried pumpkin powder conditioned at a_w_ = 0.0.

All pumpkin–inulin powders were equilibrated under various water activities in order to achieve different moisture contents. The differential scanning calorimetry thermogram of pumpkin powder conditioned at water activity equal to 0 (stored in a desiccator with phosphorus pentoxide) is shown in Figure 2. It showed a typical second-order transition. The change in the heat flow line is related to the change in the heat capacity when glass transition occurs. Similar curves were obtained for the other pumpkin powders prepared with different inulin additions. Tg values were taken as the midpoints of the transitions.

**Figure 3:**
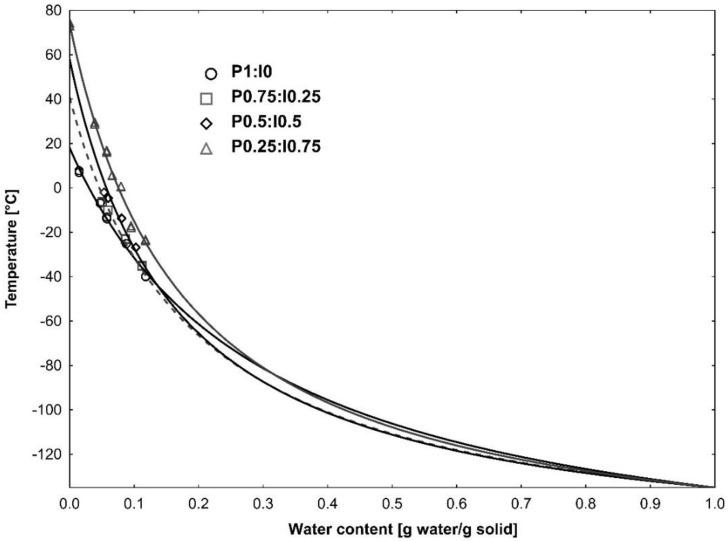
Glass transition temperature as a function of solids content for pumpkin powder produced with different inulin additions fitted by Gordon–Taylor model, P1:I0 data from ref. [9].

The relationship between the glass transition temperature and moisture content was modelled by using the Gordon–Taylor equation. The Tg values obtained for the pumpkin–inulin powders as a function of the solid fraction in the samples are shown in Figure 3. The estimated parameters of the model and coefficient of the determination values are presented in Table 2. Experimental Tg data fitted to the Gordon–Taylor equation showed satisfactory R^2^ values (above 0.990). The expected decrease of the Tg value with decreasing of the water activity values can be observed for all the powders, according to the plasticizing effect of water on the amorphous constituents of the matrix [38]. Similar observations were presented in the literature by researchers, who studied glass transition for various biopolymers, vegetables and fruits.

Table 2 shows the calculated Gordon–Taylor constants: Tgs corresponding with glass transition of anhydrous solids and the empirical K parameter. The glass transition temperatures of the plant materials is influenced by many factors, such as the water content in the product and the chemical composition and molecular weight of the solid substances [5]. In general, fruits usually showed low values of Tg because of the high concentration of low molecular weight substances, including organic acids and sugars. The low values of glass transition temperature are especially problematic in the processing and storage of foodstuffs in powdered form due to the occurring of undesirable changes such as caking, agglomeration and stickiness. In order to avoid it in food powders, high molecular weight polymers are used as a carrier agent. As expected, the addition of inulin increased the estimated Tgs value of pumpkin powder from 18.1 °C to 74.1 °C (P0.25:I0.75 sample). This observation confirms that that inulin can be considered as a carrier agent, helpful for improving the properties and stability of the food powder during storage and processing. Similar results were reported for dried blueberry juice with inulin addition [39].

According to Gordon & Taylor [21], the K parameter controls the degree of curvature of Tg dependence on water concentration in binary systems and express the strength of the interaction between components of the material. The highest value of k parameter was obtained for the sample containing 50% of pumpkin and 50% of inulin.

### 3.3. State Diagram

**Figure 4:**
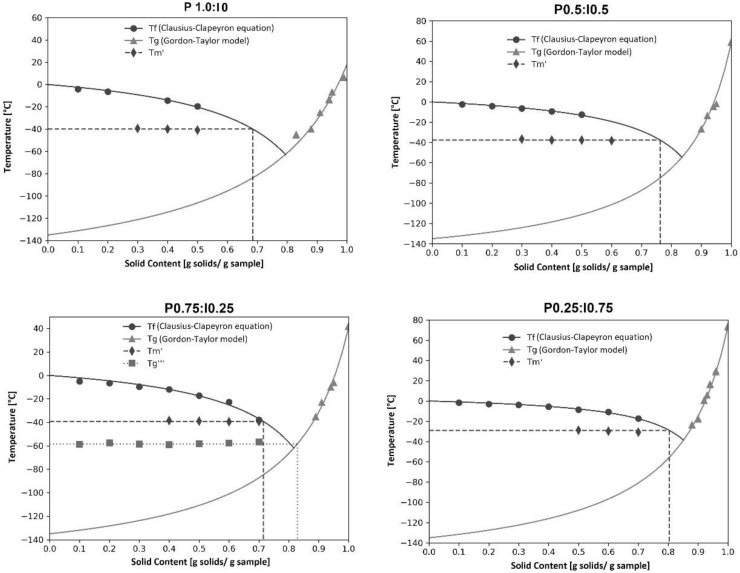
State diagrams of pumpkin–inulin freeze-dried pumpkin, P1:I0 data from ref. [9].

State diagrams are often referred to as a map showing different physical states of food as a function of solid mass or water content and temperature, which provides information about the complete state/phase changes occurring in the amorphous materials. Critical parameter values determined by using the state diagrams can therefore be very useful in the industry during the formulation of food products, the selection of appropriate technological process conditions, and at the food packaging stage. The shelf life and stability of foodstuffs can be also predicted using the state diagrams [5]. Due to the different composition and complex interactions between food ingredients, state diagrams are constructed based on data obtained through experimental analysis including mainly the determination of the freezing curve, the glass transition line and the conditions of the maximal-freeze-concentration. The state diagrams of the freeze dried pumpkin powder produced with different inulin additions are shown in Figure 4. The Gordon–Taylor model was used to plot the glass transition line and Clausius–Clapeyron equation to express the freezing curve. Because the Clausius-Clapeyron equation is limited to the ideal, for example very dilute solutions, the experimental data of the freezing curve were also fitted with its extended version of the-Chen model in order to show a better characterization of the changes in the material.

Constant, molecular weight and proper coefficient of the determination values calculated by non-linear regression and data appointed from the state diagrams are shown in Table 3. The models parameter E is defined as the molecular weight ratio of water and solids. Values of E calculated from the Chen model range between 0.085 and 0.265 and from Clausius–Clapeyron equation between 0.079 and 0.217. For most of the analyzed blends, values of E estimated from the Clausius-Clapeyron equation were lower than those calculated from the Chen model. It was found that for both used equations, the value of parameter E increased with the decreasing polymer content in the sample. The exception was the E value determined for the powder with the highest inulin content, which was slightly higher than that calculated for the sample with equal amounts of pumpkin and fructan. A similar trend was also reported for pumpkin powder produced with different maltodextrin additions [9].

Based on the values of parameter E calculated for the Chen model and the Clausius-Clapeyron equation, the molecular weight of the tested blends was determined and ranged from 68 to 206 g/mol and from 83 to 229 g/mol, respectively. In both cases, as predicted, an increase in the MW value was noted with an increasing proportion of inulin in the sample (with one exception for calculation by the Chen model). The molecular weight values determined for pumpkin-inulin powders were relatively low compared to those calculated for other food products, which may result from insufficient fitting of the model to experimental data. The values of the coefficient of determination calculated for the Chen model were slightly higher than those determined for the Clausius-Clapeyron equation. However, in the case of the Chen model, some of the estimated parameters were not statistically significant. Therefore, state diagrams plotted with curves determined using the Clausius-Clapeyron equation. Critical parameter values estimated from state diagrams of pumpkin–inulin systems were shown in Table 4. The Tm′ parameter is defined as the end point of freezing and it is known that below Tm′ the sample cools without forming ice [40]. In our research the Tm′ point was plotted from the cooling curve by the method proposed by Rahman et al. [41].

The Tm′ values estimated for pumpkin–inulin powders varied between −40.0 and −28.9 °C, which was similar to the values calculated to other dried products rich in carbohydrates such as the Chinese gooseberry [42], raspberry [43], and dates [44]. It was observed that the value of Tm′ increased with the increasing inulin content in the sample. The highest value (−28.9 °C) was determined for powders containing a 75% addition of fructan. The value of Tm′ is particularly important when setting the storage conditions for frozen food, as it as can be related to solute and solvent (water) crystallization and recrystallization during food storage [45]. The ultimate maximal-freeze concentration glass transition temperature (Tg′) and the corresponding solids content (Xs)Tg′ was estimated as a intersection of a vertical extrapolation from cross point of Tm′ line and the freezing line on the glass transition curve. In practical terms, the Tg′ value corresponds to the safe storage temperature of the material in the frozen state below which no phase transformations take place in the product. Among the two-component systems, the lowest values of Tg′ and (Xs)Tg′ were found for the powder containing the lowest dose of inulin, and this value increased with increasing fructan additions. The maximal-freeze concentration glass transition temperature calculated for binary systems varied between −84.7 °C and −55.7 °C. Comparing these results to our recent study using maltodextrin, it can be observed that the same addition of inulin raises the Tg′ value slightly less than was the case with starch hydrolysates. For example, the Tg′ determined for the sample containing 50% maltodextrin was −70.5 °C [9], while the same amount of inulin provided a Tg′ value of −75.0 °C. Such a small difference between the two carriers used indicates that with proper formulation optimization, inulin can successfully replace maltodextrin as an additive to prevent the occurrence of glass transition in frozen material. The lowest temperature value of the ultimate maximal-freeze concentration glass transition temperature was calculated for pure pumpkin powder without any addition. Compared to other foods, the value obtained is quite low because the Tg′ determined for dried tomato was −59 °C [46], dried pineapple was −51.6 °C [47], and freeze-dried plums was −57.5 °C [48]. The Tg″ value was defined as the intersection of the freezing curve to the glass line and corresponding to it (Xs)Tg″ values were also shown in Table 4 and it was similar to analogous values calculated from state diagrams of other plant materials [42,49]. Tg‴ is defined as the glass transition temperature of the solids matrix in the frozen sample, which is determined by the calorimetric technique below the Tm′ values [50]. For the tested powders, determination of the Tg‴ value was only possible for the powder containing 75% inulin addition, and it was found to be −58.5 °C. A similar value of this parameter (Tg‴ equal to −60.8 °C) was calculated for the Chinese gooseberry [42].

## 4. Conclusions

The state diagrams for pumpkin–inulin systems was developed by determining the glass line, freezing curve and maximal-freeze-concentration conditions. The addition of inulin into the pumpkin powder notably increased the glass transition temperature values, which indicates that inulin can be considered as a potential alternative to common carriers that are used in the drying processes of fruit. The initial glass transition temperature of freeze dried pumpkin increased from −40.0 °C to −28.9 °C as a result of the addition of 75% fructan. The low Tg′ value observed for pure pumpkin pulp, attributable to the high concentration of low molecular weight substances, indicates that its safe frozen storage would require maintaining a very low temperature to preserve the material in its glass-like state. A 50% addition of inulin resulted in an increase in glass transition temperature at maximum freeze-concentration of nearly 10 °C. The ultimate maximum-freeze-concentration conditions of the tested powders varied between 0.79 to 0.85 g solid/g sample and increased with the increasing amount of the fructan in the material. As expected, the addition of inulin to powdered and freeze-dried pumpkin resulted in a decrease in the hygroscopicity of the material but only in the second and third adsorption ranges (at water activities higher than 0.4). Remarkably, similar to the use of maltodextrin as a carrier, a gradual increase in the proportion of fructan in the sample resulted in a decrease in the monolayer capacity. In conclusion, it can be stated that inulin can be an alternative carrier for commonly used polymeric substances with the proper choice of additive and storage conditions such as water activity, temperature and humidity. The state diagram is a useful tool to show the mutual relationships between these parameters. In order to optimize the formulation and to determine the ideal storage conditions for two-component powders containing inulin and pumpkin, future consideration should be given to combining the data determined from the state diagram with the monolayer capacity determined from an appropriate sorption model. 

## Figures and Tables

**Table 1:** Parameters of sorption models fitted to experimental data of the pumpkin–inulin powders.

	P1:I0	P0.75:I0.25	P0.5:I0.5	P0.25:I0.75
**BET**				
**Xm**	0.100 *	0.078	0.061	0.053
**C**	1.62 *	2.92	6.83	18.58
**R^2^**	0.986 *	0.986	0.984	0.992
**RMS [%]**	8.56 *	7.12	5.56	3.50
**a_w_ range**	0.11–0.52			
**GAB**				
**Xm**	0.079 *	0.068	0.058	0.057
**C**	2.36 *	3.76	7.99	16.52
**K**	1.050 *	1.034	1.009	0.919
**R^2^**	0.998 *	0.966	0.997	0.998
**RMS [%]**	9.80 *	6.72	4.46	3.03
**a_w_ range**	0.11–0.75			
**Peleg**				
**A**	0.204	0.586	0.140	0.126
**B**	1.050	4.889	0.662	0.513
**D**	0.773	0.170	0.456	0.273
**E**	5.165	0.864	4.924	4.749
**R^2^**	0.998	0.998	0.999	0.999
**RMS [%]**	14.11	6.60	4.28	2.81
**a_w_ range**	0.11–0.86			
**Lewicki**				
**F**	0.508	0.135	0.116	0.125
**G**	0.288	0.673	0.641	0.472
**H**	6.100	1.237	0.692	0.463
**R^2^**	0.998	0.995	0.997	0.997
**RMS [%]**	10.61	9.95	6.18	3.24
**a_w_ range**	0.11–0.86			

* data from ref. [9].

**Table 2:** Parameters of the Gordon–Taylor equation fitted to experimental data of the pumpkin–inulin powders.

	P1:I0	P0.75:I0.25	P0.5:I0.5	P0.25:I0.75
**Tgs [°C]**	18.1 *	40.9	58.0	74.1
**k**	4.32 *	6.27	7.11	6.69
**R^2^**	0.990 *	0.995	0.994	0.992

* data from ref. [9].

**Table 3:** Constants of the Clausius –Clapeyron equation and Chen model fitted to the experimental data of the pumpkin–inulin powders.

	Chen Model				Clausius-Clapeyron Equation		
	B	E	R^2^	MW	E	R^2^	MW
**P1:I0**	−0.287	0.265	0.993	68	0.217 *	0.984 *	83 *
**P0.75:I0.25**	−0.006	0.186	0.978	97	0.184	0.978	98
**P0.5:I0.5**	0.452	0.085	0.981	212	0.137	0.979	132
**P0.25:I0.75**	−0.062	0.087	0.991	206	0.079	0.988	229

* data from ref. [9].

**Table 4:** Parameters estimated from the state diagrams of the pumpkin–inulin powders.

	P1:I0 *	P0.75:I0.25	P0.5:I0.5	P0.25:I0.75
Tm′ [°C]	−40	−39.2	−37.6	−28.9
(Xs) Tg′	0.685	0.715	0.762	0.803
Tg′ [°C]	−83.7	−84.7	−75	−55.7
(Xs) Tg″	0.79	0.82	0.84	0.85
Tg″ [°C]	−62.8	−61.9	−54.6	−38.6
(Xs)Tg‴	-	0.83	-	-
Tg‴ [°C]	-	−58.5	-	-

* data from reference [9].

## Data Availability

Raw data available on request to authors.

## Sample Availability

Samples of the compounds are not available from the authors.
